# Olfactory function following open rhinoplasty: A 6-month follow-up study

**DOI:** 10.1186/1472-6815-8-6

**Published:** 2008-10-03

**Authors:** Hashem Shemshadi, Mojtaba Azimian, Mohammad Ali Onsori, Mahdi AzizAbadi Farahani

**Affiliations:** 1 University of Social Welfare and Rehabilitation Sciences, Department of Clinical Sciences and Speech Reconstructive Surgery, Tehran, Iran; 2 Medicine and Health Promotion Institute, Tehran, Iran; 3 Plastic and Reconstructive Surgery, Koodakyar Street, Daneshjoo Boulevard, Evin, Tehran, Iran

## Abstract

**Background:**

Patients undergoing any type of nasal surgery may experience degrees of postoperative olfactory dysfunction. We sought to investigate "when" the olfactory function recovers to its preoperative levels.

**Methods:**

In this cohort design, 40 of 65 esthetic open rhinoplasty candidates with equal gender distribution, who met the inclusion criteria, were assessed for their olfactory function using the Smell Identification Test (SIT) with 40 familiar odors in sniffing bottles. All the patients were evaluated for the SIT scores preoperatively and postoperatively (at week 1, week 6, and month 6).

**Results:**

At postoperative week one, 87.5% of the patients had anosmia, and the rest exhibited at least moderate levels of hyposmia. The anosmia, which was the dominant pattern at postoperative week 1, resolved and converted to various levels of hyposmia, so that no one at postoperative week 6 showed any such complain. At postoperative week six, 85% of the subjects experienced degrees of hyposmia, almost all being mild to moderate. At postoperative six month, the olfactory function had already reverted to the preoperative levels: no anosmia or moderate to severe hyposmia. A repeated ANOVA was indicative of significant differences in the olfactory function at the different time points. According to our post hoc Benfronney, the preoperative scores had a significant difference with those at postoperative week 1, week 6, but not with the ones at month 6.

**Conclusion:**

Esthetic open rhinoplasty may be accompanied by some degrees of postoperative olfactory dysfunction. Patients need a time interval of 6 weeks to 6 months to fully recover their baseline olfactory function.

## Background

Since the joy of foods and/or beverages is highly correlated with their odor, any degree of olfactory dysfunction negatively impacts one's sense of well-being and content [1,2]. Not only do olfactory impairments alter one's appetite, body weight, psychological well-being, and quality of life [3], but they also compromise one's safety when a certain odor should raise the alarm in such different cases as spoiled food, leaking natural gas, or airborne pollutants [4]. Indeed, during fire alarms, those with a more intact sense of smell can perceive danger earlier and flee the scene more swiftly [5]. In short, loss or distortion of the olfactory function can even lead to mortality [6,7].

Patients undergoing open rhinoplasty may sustain postoperative olfactory complications; it should come as no surprise that olfactory impairments are a great concern for the surgeon and/or the patient [8,9]. Determining a timetable for the recovery of the olfactory function can, therefore, pave the way for a better communication between the surgeon and the patient [10] and enhance the latter's trust in esthetic and/or reconstructive nasal surgery [11].

Unfortunately, there is a dearth of data in the existing medical literature on olfactory impairments after open rhinoplasty, but we do know that surgical trauma, mucosal swelling, olfactory nerve paralysis and blood clots within the nasal cavity are most contributing factors [12,13].

We sought to determine the time required for the olfactory function to revert to its preoperative levels after open rhinoplasty.

## Methods

### Setting

Grant was awarded by the University of Social Welfare and Rehabilitation Sciences upon the approval of the study protocol by the university's Ethics Committee. Written informed consent was obtained from all the patients. Between the years 2003 and 2006, 65 candidates for open rhinoplasty in Pasargad General Hospital, Tehran, Iran were assessed for inclusion criteria.

### Participants

All the 65 patients underwent a thorough past and present medical history check and physical examination. The patients' nasal mucous membranes were specifically evaluated for any dryness, leukoplakia, and exudates to check for any signs of inflammations. Routine blood analysis, chest X-ray, and computerized tomography of paranasal sinuses (coronal and plain) were performed in all the patients preoperatively, all of which were within normal limits [14]. Patients with any previous surgical operations, systemic diseases, interfering medications, smoking history, intranasal substance abuse (e.g. cocaine), psychological or psychiatric instability, and need for more extensive nasal surgery (e.g. sub-mucosal septal cartilage resections or inferior conchal cauterization) were excluded. A minimum level of high school diploma was required for patient selection in order to facilitate cooperation [15,16]. Of the initial 65 patients, 40 met the inclusion criteria and were included in the study. None of these subjects showed significant post operative complications, whether early such as hemorrhage and acute infection or late such as chronic infection, septal perforation, and nasal obstruction.

### Intervention

All the patients underwent almost a similar approach to open rhinoplasty by the same surgeon (first author) and same monitored (standby) anesthesia. After opening via columellar v-incision, dorsal hump removal and bilateral osteotomies along with caudal septum resection for columellar, anterior maxillary grafts and tip refining were performed. There was no need for more any other intranasal touches [17]. Preoperatively, intranasal mesh packing of 1 ml xylocaine10% mixed with 1 ml Phenyephrine 0.5% for 1 minute and postoperatively, intranasal half-sheet Vaseline gauze mixed with 10 gm Achromycin packing were applied for 48 hours.

### Main outcome and follow-up

The olfactory function was measured as the main outcome preoperatively and postoperatively at week1, week 6, and month 6. The olfactory function was assessed using the Smell Identification Test (SIT), which is based on a booklet containing 40 numbered pages, with four-choice questions on each page for the 40 participants [18-20]. Forty culturally well-known odors were selected (Table 1). Dark, odorless, numbered, 250 ml, label-less sniffing bottles produced by "Ghamsar Kashan Takgol Co., Ltd." were employed, each bottle being associated with the 40 numbered pages in the booklet. The subjects were asked to identify each odor using the sniffing bottles and select a response among the four choices given in each page, within twenty minutes [21]. The patients were also requested to give no response, gaining no credit, if they failed to recognize an odor. The olfactory function was classified into normosmia, hyposmia, and anosmia. Anosmia, as a complete lack of the olfactory function, was defined as a SIT score < 20. Hyposmia, defined as a composite test score of 20–35, with further dividing into mild, moderate, and severe in respect to test scores of 31–35, 26–30, and 21–25. Normosmia, as the normal olfactory function, was defined as scores > 35 [22,23]. The same cut points were considered for both female and male subjects.

**Table 1:** 40 culturally well-known Iranian odors produced by "Ghamsar Kashan Takgol., Co. Ltd."

**In alphabetical order**	
Ajowan (zenian)	Jasmine (yas)
Birds foot (shanbalilah)	Juglans regia (barg gerdoo)
Black coffee (ghahvah)	Leek (tarah)
Black tea (chaee)	Lemon balm (badranjobah)
Chamomile (baboonah)	Matricaria (baboonah)
Cardamom (hel)	Medicago sativa (yonjah)
Chicory (kasni)	Olea europaea (barg zaytoon)
Cinnamon (darcheen)	Onion (piaz)
Clove (mikhak)	Orange flower (baharnarenge)
Coconut (nargeel)	Outsole (zirah)
Coriander (geshniz)	Pulegium vulgare (pooneh)
Dill (sheveed)	Rosa canina (nastaran)
Eucalyptus (okaliptoos)	Rose leaves (golab)
Foeniculum (razianah)	Salix capraea (bidmeshk)
Fumaria (shahtarah)	Satureia hortensis (marzah)
Garlic (seer)	Spearmint (naena)
Borago officinalis (golgavzaban)	Valeriane officinalis (sonbolteep)
Ginger (zangafil)	Vanilla (barg vanil)
Glycyrrhiza glabra (shirinbayan)	Vinegar (serkeh)
Hysope (barg zoofa)	Zizyphora tenuior (kakooti)

Parentheses are the Persian version of the odors used in the smell identification tests.

### Statistical analysis

All the statistical analysis were carried out using the SPSS program. The significance of the differences between the SIT scores at the different time points were measured with a repeated ANOVA. Possible differences in the time points were determined via a post hoc Benfronney test. Gender was considered as a factor in this analysis so as to determine its possible effect on the SIT changes. A P-value < 0.05 was considered statistically significant.

## Results

### Participants

From the initial 65 esthetic open rhinoplasty candidates, 40 persons at a mean age of 25.0 years (range: 20–30, SD = 3.1 years) met our inclusion criteria and were included in our study. The study population was comprised of 20 (50%) men and 20 (50%) women.

### SIT scores

#### Different hyposmia levels at different time points

The preoperative frequency of SIT scores < 20 was 0 (0%).

The frequencies of SIT scores < 20 were 35 (87.5%) at week 1; 0 (0%) at week 6 and 0 (0%) at month 6, postoperative.

**Figure 1:**
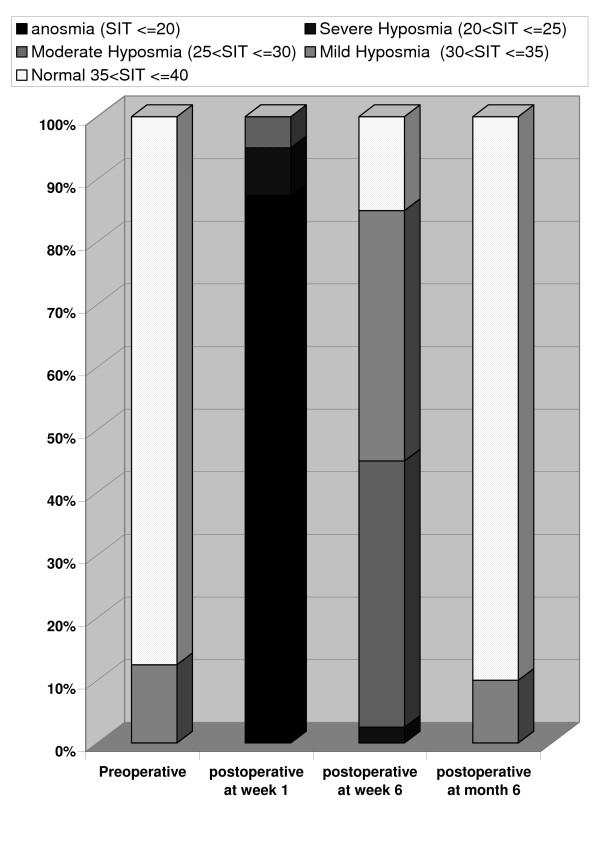
Frequency of normosmia, hyposmia, and anosmia in 4 stages: from preoperative stage to postoperative month 6.

The preoperative frequency of SIT scores < 30 was 0 (0%). The postoperative frequencies of SIT scores < 30 were 40 (100%), 18 (45%), and 0 (0%) at week 1, week 6, and month 6, respectively (Table 2, Figure 1).

**Table 2:** Frequencies of levels of olfactory function according to Smell Identification Test (SIT) scores following open rhinoplasty

	Anosmia (SIT ≤ 20)	Severe hyposmia (20 < SIT ≤ 25)	Moderate Hyposmia (25 < SIT ≤ 30)	Mild Hyposmia (30 < SIT ≤ 35)	Normal 35 < SIT ≤ 40
Preoperative	0 (0%)	0 0%)	0 (0%)	5 (12.5%)	35 (87.5%)

postoperative week 1	35 (87.5%)	3 (7.5%)	2 (5%)	0 (0%)	0 (0%)

postoperative week 6	0 (0%)	1 (2.5%)	17 (42.5%)	16 )40%)	6 (15%)

postoperative month 6	0 (0%)	0 (0%)	0 (0%)	4 (10%)	36 (90%)

#### Comparison between pre and postoperative SIT scores

**Figure 2:**
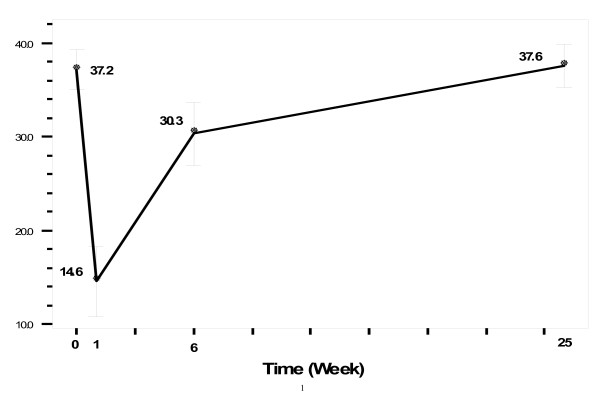
Mean (SD) SIT scores in 4 stages: from preoperative stage to postoperative month 6.

Our repeated ANOVA showed significant differences between the olfactory function scores at the different time points. According to the post hoc Benfronney, these differences were between the preoperative scores and postoperative ones at week 1, week 6, and month 1, but not between the preoperative scores and those at postoperative month 6. Gender did not affect the changes between the different time points (Figure 2).

## Discussion

The olfactory function shows a temporary decline following open rhinoplasty but tends to revert to its preoperative levels 6 months post surgery. This may be a reassuring answer to candidates for this kind of nasal surgery since they are likely to inquire whether the loss or decrease in their sense of smell is reversible, and, if reversible, how long it is before their sense of smell is normal again [24].

One week after open rhinoplasty, anosmia was present in 87.5% of our patients, and the rest exhibited moderate to severe hyposmia. In other words, almost all the patients were anosmic shortly after open rhinoplasty. By postoperative week 6, however, anosmia had downgraded to mainly mild to moderate levels of hyposmia. All our patients had regained their preoperative olfactory function levels by postoperative month 6; most were normosmic and a few were mildly hyposmic.

In a study of 93 patients undergoing various types of nasal surgery, including ethmoidectomy, polypectomy, Caldwell-Luc procedure, open reduction of nasal fracture, closed reduction of nasal fracture, rhinoplasty, and septoplasty, 34% of the patients experienced degrees of decline in their olfactory function. In 1% of the patients, anosmia persisted for a long time [15]. In a review of 200 rhinoplasty patients, Champion [25] noted that 10% complained of temporary anosmia.

The reason for the differences between the results of previous studies and those in the present study can be the different types of nasal surgery covered in the investigations: while some procedures may cause direct trauma to the olfactory neuroepithelium itself or distortion of the intranasal anatomy, some surgeries such as esthetic open rhinoplasty have a very mild or even no such direct impacts.

Our results, demonstrating a complete return of the olfactory function to the baseline values, do not chime in with those in the Heywood et al. study, which showed mainly incomplete degrees of improvement [22].

It is noteworthy that among the authors who believe that the olfactory function will return to its pre-surgery values, there is no consensus regarding the time required for this improvement. Indeed, such different time periods as less than 6 months [26], a minimum of 6 months, [27], and longer periods up to 18 months [25] have been mentioned. Indubitably, however, a meticulous preoperative medical history check and physical examination in conjunction with proper paraclinical tests can not only determine the preoperative status of the olfactory function but also enable the surgeon to provide the patient with more definite answers as regards queries about time [13].

In our study, gender was not a predictor of the amount of the olfactory acuity loss; the reason may lie in the fact that our sample size was limited. In contrast to our studies, some investigators have reported gender as one of the predictors of olfactory impairment, in conjunction with some other variables such as use of general anesthesia and amount of swelling in the nasal mucous membrane [15,28].

A proportion of our patients had mild hyposmia at postoperative month 6, but all of them had this condition before the surgery as well. As these patients are not usually aware of their impaired olfaction preoperatively, they may wrongly attribute their remained distorted sense of smell to the surgical procedure. Documentation of the preoperative olfactory function before nasal surgery can preclude such postoperative claims [1].

Olfaction is susceptible to temporary disorders, following a congestion or edema, as the olfactory nerve fibers cross through multiple small foramina in the cribriform plate of ethmoid and crista galli [4]. These indirect causes may also include indirect disturbances engendered by pharmacologic agents or mucosal edema or other processes, hence the general rule: "All types of nasal surgery can potentially worsen the olfactory function" [15], even in the absence of direct trauma to the olfactory nerve or even nasal mucosa [5]. External cosmetic rhinoplasty is no exception.

The present study had some limitations. The sample size was small, and there was no access to any published data on the sensitivity and normal ranges of the Iranian population with respect to the smell test utilized. Another drawback of the present study was the fact that odor identification is strongly dependent on familiarity with these odors [29], and it is essential that culture be taken into consideration when developing smell tests specific for each country [23]. As result, a modified smell test had to be used for our samples, all of whom were Iranian. Although our subjects' high preoperative scores attest to our meticulous selection of relatively identifiable (and pleasant) odors, this use of a modified smell test precluded a comparison between our results and those reported previously. The present study did not investigate the possible predictors of postoperative olfactory change, and nor did it include variables such as "medications", "durations of packing period", "procedures", and "duration of decreasing gross inflammation".

## Conclusion

The temporary decline in the olfactory function following open rhinoplasty can be expected to fully resolve by postoperative month 6.

## Pre-publication history

The pre-publication history for this paper can be accessed here:


